# Corrigendum to “Seroprevalence of Dengue IgG Antibodies among Healthy Adult Population in Lahore, Pakistan”

**DOI:** 10.1155/2017/6138754

**Published:** 2017-11-22

**Authors:** Shahid Mahmood, Hibbah Nabeel, Saadia Hafeez, Urooj Zahra, Hammad Nazeer

**Affiliations:** ^1^Department of Community Medicine, Gujranwala Medical College, Gujranwala, Pakistan; ^2^Fatima Jinnah Medical College, Lahore, Pakistan; ^3^Department of Infectious Diseases, Shaukat Khanum Memorial Hospital, Lahore, Pakistan

In the article titled “Seroprevalence of Dengue IgG Antibodies among Healthy Adult Population in Lahore, Pakistan” [1], the name of the second author was given incorrectly as Hiba Nabeel. The author's name should have been written as Hibbah Nabeel. The revised authors' list is shown above.
